# The reliability and validity of a Japanese version of symptom checklist 90 revised

**DOI:** 10.1186/1751-0759-2-19

**Published:** 2008-10-28

**Authors:** Mitsunao Tomioka, Midori Shimura, Mikio Hidaka, Chiharu Kubo

**Affiliations:** 1 Department of Psychosomatic Medicine, Kyushu University Hospital, 3-1-1 Maidashi, Higashi-ku, Fukuoka City, 812-8582, Japan; 2 Karibe CLINIC, SOTSU BLD. 2F, 1-3 Honmachi, Naka-ku, Yokohama City, 231-0005, Japan; 3 Faculty of Literarure, Kurume University, 1635 Mii-machi, Kurume City, Fukuoka Prefecture, 839-8502, Japan

## Abstract

**Objective:**

To examine the validity and reliability of a Japanese version of the Symptom Checklist 90 Revised (SCL-90-R (J)).

**Methods:**

The English SCL-90-R was translated to Japanese and the Japanese version confirmed by back-translation. To determine the factor validity and internal consistency of the nine primary subscales, 460 people from the community completed SCL-90-R(J). Test-retest reliability was examined for 104 outpatients and 124 healthy undergraduate students. The convergent-discriminant validity was determined for 80 inpatients who replied to both SCL-90-R(J) and the Minnesota Multiphasic Personality Inventory (MMPI).

**Results:**

The correlation coefficients between the nine primary subscales and items were .26 to .78. Cronbach's alpha coefficients were from .76 (Phobic Anxiety) to .86 (Interpersonal Sensitivity). Pearson's correlation coefficients between test-retest scores were from .81 (Psychoticism) to .90 (Somatization) for the outpatients and were from .64 (Phobic Anxiety) to .78 (Paranoid Ideation) for the students. Each of the nine primary subscales correlated well with their corresponding constructs in the MMPI.

**Conclusion:**

We confirmed the validity and reliability of SCL-90-R(J) for the measurement of individual distress. The nine primary subscales were consistent with the items of the original English version.

## Background

The Symptom Checklist 90 Revised (SCL-90-R) developed by L.R. Derogatis [1] has been widely used as a clinical assessment and research tool in many countries and is cited in many articles. The scale can assess nine primary psychological symptoms as follows: Somatization (SOM), Obsessive-Compulsive (O-C), Interpersonal Sensitivity (INT), Depression (DEP), Anxiety (ANX), Hostility (HOS), Phobic Anxiety (PHOB), Paranoid Ideation (PAR), and Psychoticism (PSY).

Although this scale was developed in the late 1970's [2,3], it can still be helpful for understanding psychiatric diseases as classified in the latest version of the diagnostic criteria of DSM-IV[4] and ICD-10 [5].

Many studies have reported that a patient presenting one functional somatic disorder also has symptoms related to psychiatric disorders. For example, Juang et al. [6] reported comorbidity of depressive and anxiety disorders in chronic headache. Seventy-eight percent of patients with migraine had psychiatric comorbidity, including major depression (57%), dysthymia (11%), panic disorder (30%), and generalized anxiety disorder (8%). Sixty four percent of patients with chronic tension-type headache had psychiatric comorbidity, including major depression (51%), dysthymia (8%), and panic disorder (22%). Comorbidity of major depression and panic disorder has often been reported [7,8].

It has been shown that some medicines are effective against multiple psychiatric diseases. For example, selective serotonin reuptake inhibitors (SSRI) are antidepressants that can also be effective against obsessive-compulsive disorder [9], panic disorder [10] and social anxiety disorder [11].

Multi-dimensional assessment tools for psychiatric conditions are needed for the diagnosis of patients in psychosomatic or psychiatric settings.

The only inventory that includes most aspects of emotional disturbances within a comprehensive classification of psychiatric diseases in Japan is the Minnesota Multiphasic Personality Inventory (MMPI)[12]. However, this scale can become a considerable burden to patients because there are a great many questions. More compact scales for measuring multiple psychiatric dimensions are needed.

In this paper we report the development of a Japanese version of SCL-90-R (SCL-90-R(J)). In study 1 the original English version of the SCL-90-R was translated into Japanese and factorial validity and internal consistency were calculated to confirm reliability. Study 2 was done to determine the test-retest reliability of the SCL-90-R(J) by testing psychosomatic medicine outpatients and healthy persons. The aim of study 3 was to investigate the relationship between SCL-90-R(J) and existing scales currently used to measure psychological symptoms in Japan. This investigation was done to show the convergent-discriminant validity of the primary nine subscales of SCL-90-R(J).

## Methods

### Subjects

The SCL-90-R(J) was distributed to 1,200 people living in the suburbs of Fukuoka City who reported for a free medical examination given by the municipal government. The completed forms were returned by mail. Available for analysis were 460 complete forms of 507 (42%) residents who replied (143 men, average age 59.6 years, and 317 women, average age 50.9 years) in study 1.

118 patients visiting the Department of Psychosomatic Medicine at Kyushu University Hospital who gave informed consent to the study and were given guarantees of protection of private data in the possession their doctor. The SCL-90-R(J) was administered at one visit, and a retest was administrated at the next visit. Available for analysis were 104 inventories completed within 35 days (34 men, average age 39.4 years, and 70 women, average age 40.0 years) in study 2. As for the primary diseases of 104 patients, 70.2% were mental diseases and 29.8% were physical diseases. When classified by the mental diseases mentioned in the patients' medical record, 40.4% were diseases related to depression, including diagnosis of depression, neurotic depression, dysthymic disorder. 22.1% were diseases related to anxiety, including anxiety disorder, panic disorder, posttraumatic stress disorder (PTSD), and anxiety neurosis. 4.8% were somatoform disorders, including pain disorder, body dysmorphic disorder and psychogenic aphonia. Two patients had eating disorders and one patient had sleep disorder. As for physical diseases, 13.5% were vegetative syndrome, 6.7% were irritable bowel syndrome, and 2.9% were bronchial asthma. Atopic dermatitis and hyperventilation syndrome were 1.9% each. Functional dyspepsia, hypertendion and combined headache were suffered by one patient each.

In study 2, also analyzed were the SCL-90-R(J) test/retest results of 124 healthy undergraduate students (28 men, average age 19.3 years, and 96 women, average age 19.1 years) who attended a psychological guidance lecture at a university in Fukuoka prefecture. The period between test and retest was a week.

Eighty-seven inpatients at Kyushu University Hospital Department of Psychosomatic Medicine replied to the SCL-90-R(J). Of these patients, the results of 80 who replied to both the SCL-90-R(J) and MMPI were analyzed in study 3 (26 males, mean age 37.8 years (SD = 11.7), and 54 females, mean age 33.0 years (SD = 16.1)). Five of eighty patients were overlapped with outpatients who were employed for test/retest examination. As for the primary diseases of 80 patients, 70% were mental diseases and 30% were physical diseases. As classified by DSM-IV, 26.3% were depressive disorders, 21.3% were eating disorders, 11.3% were anxiety disorders, 5.0% were somatoform disorders, 3.8% were adjustment disorders and 2.5% were sleep disorders. As for physical diseases, 16.3% (N = 13) were diabetes mellitus (DM). Ten diabetics were Type 1 DM, and all patients had eating disorders. 3.8% had bronchial asthma and 2.5% had irritable bowel syndrome. Atopic dermatitis, lactose intolerance, cough variant asthma, Crohn's disease, spasmodic torticollis, and chronic fatigue syndrome were experienced by one patient each.

### SCL-90-R(J)

Permission was obtained from the copyright holder to translate the original version of SCL-90-R to Japanese. The translation to Japanese was done by two psychologists. The Japanese version was then back-translated to check for correspondence with the English version. The respondents were asked to describe how much each of 90 problems had distressed or bothered them in the past seven days, including that day. The descriptors were as follows: not at all (0), a little bit (1), moderately (2), quite a bit (3), extremely (4).

### MMPI

The MMPI is often used in Japan to assess the psychological state and is commonly used in other countries. Studies of the reliability and validity of this scale have been reported in many articles. To validate the SCL-90-R(J), not only the 10 standard clinical scales (C), but also Wiggins [13] content scales (W) and Tryon's [14] cluster scales (T) were scored.

### Data analysis

The Statistical Analysis System (SAS) was used for data analysis. In study 1, confirmative factor analysis was done by the Calis Procedure for covariance structure analysis. Cronbach's alpha coefficients were calculated by Corr Procedure to examine the internal consistencies of each of the nine subscales. In study 2, Pearson's correlation coefficients between the first and second test administrations were calculated using the Corr Procedure to examine the test-retest reliability. In study 3, Pearson's correlation coefficients between subscale scores of SCL-90-R(J) and MMPI scores were calculated.

## Results

### Factorial validity and internal consistency (Study 1)

**Figure 1:**
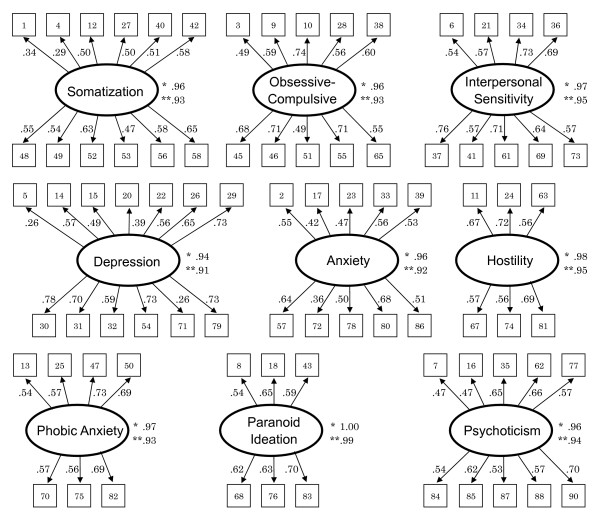
**Confirmative factor analysis of the nine primary symptom constructs of SCL-90-R(J).** Numbers in boxes accord with the item number of SCL-90-R(J), please see references [1] and [3]. *Goodness of Fit Index(GFI), **Adjusted GFI(AGFI).

The results of confirmative factor analysis are shown in Figure 1. The analysis was done for each of the nine factors. All values of the goodness of fit index (GFI) and adjusted GFI for the nine primary symptoms were over .90. Except for two items on SOM and two items on DEP, the correlation coefficients between each factor and item were over .35.

Cronbach's alpha coefficients were .76 (PHOB) to .86 (INT)(Table 1).

**Table 1:** Internal consistency and test-retest reliability coefficients of SCL-90-R(J)

	Internal Consistency (coefficient α)	Test-Retest (Pearson's r)	
	
	Community N = 460	Outpatients N = 104	Students N = 124
Somatization	.81	.90*	.73*
Obsessive-Compulsive	.85	.85*	.74*
Inter person al Sensitivity	.86	.88*	.76*
Depression	.85	.86*	.76*
Anxiety	.79	.88*	.73*
Hostility	.79	.82*	.67*
Phobic Anxiety	.76	.88*	.64*
Paranoid Ideation	.79	.83*	.78*
Psychoticism	.83	.81*	.75*

*P < .0001

### Test/retest reliability (Study 2)

Correlations between test and retest were calculated for outpatients and students (Table 2). Pearson's correlation coefficients were .81 (PSY) to .90 (SOM) for the outpatients and .64 (PHOB) to .78 (PAR) for the student group.

**Table 2:** Correlations between SCL-90-R(J) primary symptom dimensions and MMPI Clinical(C), Wiggin s(W), and Tryon-Stein-Chu (T) scales

Symptom	Correlation^1)^	Symptom	Correlation^1)^
**Somatization**		Phobia (W)	.63*
Body Symptoms (T)	.74*	Schizophrenia (C)	.60*
Hypochondriasis (C)	.70*	Psychoticism (W)	.60*
Organic Symptoms (W)	.70*	Autism (T)	.58*
Poor Health (W)	.56*	Depression (W)	.57*
		Organic Symptoms (W)	.56*
**Obsessive-Compulsive**		Poor Morale (W)	.54*
Psychasthenia(C)	.68*	Depression (T)	.52*
Tension (T)	.68*	Hypochondriasis (C)	.51*
Autism (T)	.67*		
Schizophrenia (C)	.65*	**Hostility**	
Depression (W)	.61*	Manifest Hostility (W)	.67*
Poor Morale (W)	.61*	Suspicion & Mistrust (T)	.61*
Psychoticism (W)	.60*	Resentment & Aggression (T)	.59*
Organic Symptoms ((W)	.60*	Depression (W)	.57*
Body Symptoms (T)	.57*	Psychoticism (W)	.57*
Phobias (W)	.56*	Tension (T)	.57*
Depression (T)	.56*	Schizophrenia (C)	.55*
Resentment & Aggression (T)	.55*	Depression (T)	.55*
Hypochondriasis (C)	.52*	Poor Morale (W)	.54*
Manifest Hostility (W)	.51*	Psychasthenia (C)	.53*
Hypomania (W)	.51*		
		**Phobic Anxiety**	
**Interpersonal Sensitivity**		Psychoticism (W)	.57*
Poor Morale (W)	.72*	Tension (T)	.55*
Psychasthenia(C)	.70*	Phobias (W)	.52*
Depression (W)	.68*	Depression (W)	.51*
Depression (T)	.68*	Schizophrenia (C)	.50*
Psychoticism (W)	.65*	Suspicion & Mistrust (T)	.50*
Suspicion & Mistrust (T)	.65*		
Schizophrenia (C)	.64*	**Paranoid Ideation**	
Tension (T)	.63*	Psychoticism (W)	.62*
Autism (T)	.62*	Schizophrenia (C)	.61*
Manifest Hostility (W)	.59*	Suspicion & Mistrust (T)	.61*
Resentment & Aggression (T)	.58*	Autism (T)	.59*
Phobias (W)	.54*	Psychasthenia (C)	.57*
		Depression (W)	.56*
**Depression**		Poor Morale (W)	.56*
Psychasthenia (C)	.78*	Manifest Hostility (W)	.53*
Depression (W)	.76*	Depression (T)	.53*
Depression (T)	.71*	Resentment & Aggression (T)	.52*
Tension (T)	.71*	Authority Conflict (W)	.51*
Schizophrenia (C)	.70*	Tension (T)	.51*
Poor Morale (W)	.70*	Hypomania (W)	.50*
Autism (T)	.64*		
Psychoticism (W)	.62*	**Psychoticism**	
Resentment & Aggression (T)	.60*	Psychoticism (W)	.69*
Phobias (W)	.59*	Suspicion & Mistrust (T)	.67*
Body Symptoms (T)	.58*	Schizophrenia (C)	.66*
Organic Symptoms (W)	.54*	Autism (T)	.65*
Manifest Hostility (W)	.54*	Psychasthenia (C)	.64*
Suspicion & Mistrust (T)	.54*	Depression (W)	.64*
Hypochondriasis (C)	.52*	Manifest Hostility (W)	.61*
Paranoia (C)	.51*	Tension (T)	.59*
Psychopathic Deviate (C)	.50*	Poor Morale (W)	.58*
		Resentment & Aggression (T)	.58*
**Anxiety**		Depression (T)	.55*
Tension (T)	.71*	Hypomania (W)	.51*
Psychasthenia (C)	.66*	Hypomania (C)	.50*

^1) ^Displayed correlation coefficient was .50 or more. N = 80. *p < 0.0001

### Convergent-discriminant validation (Study 3)

Pearson's product-moment correlation coefficients were calculated between the nine primary subscales of SCL-90-R(J) and the multidimensional subscale of MMPI (Table 2).

The SOM scale showed high correlations with Body Symptoms (T) and Hypochondriasis (C). The SOM scale showed correlation coefficients lower than 0.20 (p > .05) with Masculinity-Femininity (C), Social Introversion (C), Social Maladjustment (W), Feminine Interest (W) and Religious Fundamentalism (W).

The O-C scale showed high correlations with Pshchasthenia (C), Tension (T), and Autism (T). The O-C scale showed correlation coefficients lower than 0.20 (p > .05) with Hysteria (C), Masculinity-Femininity (C) and Feminine Interest (W).

The INT scale showed high correlations with Poor Morale (W), Psychasthenia (C), and Depression (W, T). The INT scale showed correlation coefficients lower than 0.20 (p > .05) with Hysteria (C), Masculinity-Femininity (C), Feminine Interest (W) and Religious Fundamentalism (W).

The DEP scale showed high correlations with Psychasthenia (C), Depression (W, T), and Tension (T). The DEP scale showed correlation coefficients lower than 0.20 (p > .05) with Hysteria (C), Masculinity-Femininity (C), Social Maladjustment, Feminine Interest (W) and Religious Fundamentalism (W).

The ANX scale showed high correlations with Tension (T), Psychasthenia (C), and Phobia (W). The ANX scale showed correlation coefficients lower than 0.20 (p > .05) with Masculinity-Femininity (C), Feminine Interest (W) and Religious Fundamentalism (W).

The HOS scale showed high correlations with Manifest Hostility (W), Suspicion and Mistrust (T), and Resentment and Aggression (T). The HOS scale showed correlation coefficients lower than 0.20 (p > .05) with Hysteria (C), Masculinity-Femininity (C), Feminine Interest (W) and Religious Fundamentalism (W).

The PHOB scale showed high correlations with Psychoticism (W), Tension (T), and Phobias (W). The PHOB scale showed correlation coefficients lower than 0.20 (p > .05) with Hysteria (C), Masculinity-Femininity (C), Social Introversion (C, T), Social Maladjustment (W), Feminine Interest (W), Religious Fundamentalism (W) and Family Problems (W).

The PAR scale showed high correlations with Psychoticism (W), Schizophrenia (C), and Suspicion and Mistrust (T). The PAR scale showed correlation coefficients lower than 0.20 (p > .05) with Depression (C), Hysteria (C), Masculinity-Femininity (C) and Feminine Interest (W).

The PSY scale showed high correlations with Psychoticism (W), Suspicion and Mistrust (T), and Schizophrenia (C). The PSY scale showed correlation coefficients lower than 0.20 (p > .05) with Depression (C), Hysteria (C), Masculinity-Femininity (C), Social Maladjustment (W), Feminine Interest (W) and Religious Fundamentalism (W).

### Gender differences

The scores of the nine subscales for all subjects of this study are displayed in Table 3. Gender differences are also examined in this table. In the community sample, gender differences were found in O-C (t(458) = 2.32, p = .020) and PSY (t(241) = 2.58, p = .010). In the student sample, gender difference was found in PAR (t(122) = 2.08, p = .040). For outpatients, gender differences were found in SOM (t(102) = 2.35, p = .020), O-C (t(102) = 2.09, p = .039), HOS (t(95.6) = 2.53, p = .013) and PAR (t(102) = 2.24, p = .027). For inpatients, no gender difference was found among the nine subscales.

**Table 3:** The basic data and gender differences of the nine primary scales for community, student, out patient, and inpatient samples^1)^

	SOM	O-C	INT	DEP	ANX	HOS	PHOB	PAR	PSY
Community samples									
All (N = 460)	.63 (.48)	.72 (.56)	.56 (.52)	.73 (.53)	.38 (.39)	.47 (.50)	.16 (.30)	.52 (.53)	.28 (.38)
Men (N = 143)	.64 (.50)	.81 (.59)	.54 (.49)	.75 (.49)	.43 (.41)	.52 (.50)	.16 (.32)	.59 (.55)	.36 (.42)
Women (N = 317)	.63 (.47)	.68 (.55)	.58 (.54)	.73 (.55)	.36 (.38)	.45 (.51)	.16 (.29)	.49 (.52)	.25 (.36)
*P*^2)^	n s	.020	n s	n s	n s	n s	n s	n s	.010
Undergraduate students									
All (N = 124)	.71 (.58)	.99 (.63)	.82 (.68)	.95 (.69)	.68 (.62)	.74 (.68)	.30 (.45)	.65 (.68)	.40 (.53)
Men (N = 28)	.66 (.47)	.91 (.59)	.84 (.67)	.88 (.67)	.54 (.48)	.78 (.67)	.27 (.43)	.88 (.75)	.46 (.57)
Women (N = 96)	.73 (.61)	1.02 (.64)	.82 (.69)	.97 (.70)	.72 (.65)	.72 (.69)	.31 (.46)	.58 (.64)	.38 (.52)
*P*^2)^	ns	ns	ns	ns	ns	ns	ns	.040	ns
Outpatients									
All (N = 104)	1.14 (.86)	1.28 (.91)	1.13 (.90)	1.45 (.93)	1.04 (.88)	.85 (.80)	.75 (.85)	.92 (.85)	.65 (.63)
Men (N = 34)	.86 (.74)	1.02 (.87)	.89 (.92)	1.19 (.95)	0.84 (.79)	.61 (.55)	.57 (.81)	.66 (.75)	.51 (.61)
Women (N = 70)	1.27 (.89)	1.41 (.90)	1.25 (.87)	1.57 (.91)	1.14 (.90)	.97 (.88)	.83 (.87)	1.05 (.87)	.72 (.63)
*P*^2)^	.020	.039	n s	n s	n s	.013	n s	.027	n s
Inpatients									
All (N = 80)	1.05 (.75)	1.29 (.94)	1.14 (.89)	1.60 (.96)	1.03 (.92)	.79 (.92)	.69 (.77)	.94 (.92)	.86 (.82)
Men (N = 26)	1.07 (.73)	1.28 (.94)	1.13 (.92)	1.54 (.92)	1.03 (.87)	.87 (.94)	.71 (.68)	1.04 (.95)	.83 (.89)
Women (N = 54)	1.04 (.76)	1.30 (.95)	1.14 (.89)	1.63 (.99)	1.03 (.95)	.75 (.91)	.68 (.82)	.90 (.91)	.88 (.80)
*P*^2)^	n s	n s	n s	n s	n s	n s	n s	n s	n s

SOM:Somatization, O-C: Obsessive-Compulsive, INT: Interpersonal Sensitivity, DEP: Depression, ANX: Anxiety, HOS: Hostility, PHOB: Phobic Anxiety, PAR: Paranoid Ideation, PSY: Psychoticism.

^1) ^Means (SD)

^2)^The results of t-test on gender differences (men vs women). ns: none significance

## Discussion

### The validity and reliability of SCL-90-R(J)

We examined the validity and reliability of SCL-90-R(J) for use as a psychometric scale.

SCL-90-R(J) was consistent with the nine primary scales of the original version (Study 1). The data showed that each subscale consisted of items similar enough to the English version that each scale had sufficient internal consistency, indicating its reliability for psychometric testing.

Test-retest reliability was sufficient for the outpatient group. The student group indicated moderate but lower reliability coefficients than the outpatient group (Study 2). The students may have responded according to their mood at the time they filled out the questionnaire rather than to their symptoms, while the patients responded to their symptoms. Because the retest was done by the students at a shorter interval than the patients, the results for the students were of higher reliability than those of the patients. The results were the reverse to our expectations, suggesting that this scale will show a superior performance when used for patients than for healthy persons such as students.

Convergent-discriminant validation demonstrates that measures of interest show strong correlations with independent measures of the same construct and show weak or no correlation with measures of dissimilar constructs. The primary nine subscales of SCL-90-R(J) were strongly correlated with the MMPI subscales of the same or similar construct (Study 3). In addition, dissimilar subscales of MMPI had lower correlation with the SCL-90-R(J) subscales. These results also suggest that the SCL-90-R(J) has convergent and discriminant validity and that the scale is able to assess the constructs that it is intended to measure.

Because we translated the English checklist into Japanese, it was possible that an individual item had a weak relationship with the subscale in the original version. First, we examined whether or not the nine primary scales of the Japanese version consisted of items the same as the original version (study 1). Confirmative factor analysis showed that almost all items highly correlated with each factor (symptoms) contained in the items in the original version. The data showed that our nine subscales of the Japanese version consisted of the same items as in the English original version. Confirmative factor analysis also demonstrated that each factor had high validation.

Research that would benefit from the use of SCL-90-R(J) includes comparative cross-cultural studies. Several studies have been published that have compared the psychopathology between countries using the SCL-90-R [15,16]. These studies need to be consistent in their use of the same scale from the same items. Our version has this necessary condition with good validation.

Convergent-discriminant validation was demonstrated by contrasting the subscale scores of SCL-90-R(J) with scores from the MMPI. As summarized in Table 2, nine subscales were consistent with the corresponding scales from MMPI. The tendency of the correlation coefficients was similar to the data of Derogatis et al. [17]. In their article, they reported that O-C was not well correlated with MMPI constructs because there was no directly comparable scale on the MMPI. Although this was not changed essentially in our design, the O-C subscale had a moderate relationship with the subscale from MMPI, which would explain the clinical symptoms of obsessive-compulsive disorder in our results.

SCL-90-R is a good tool for researching individual psychological distress. Because this psychological scale can evaluate multidimensional psychopathological aspects simultaneously, it is effective as a screening test for medical examination and treatment. It is also effective for an effect judgment of drug efficacy, such as SSRI against anxiety symptoms [10,11] as well as a depressive mood [18,19]. For example, co-morbidity of major depression and panic disorder has often been reported [7,8], which indicates that SCL-90-R(J) is suitable for the evaluation of patients having such plural diseases. This scale would also be useful for researching the correlation between physical diseases and psychological dysfunction. Mikocka-Walus et al. [20] used this scale to examine the relationship between mental distress and relapse in patients with diseases of the digestive tract.

### Gender differences

Gender differences in SCL-90-R have been reported [21-24]. Previous articles reported that there was a tendency toward higher scores for females than males on the nine subscales. Most of the gender differences on the nine subscales were statistically significant. The trend was particularly notable for the outpatient sample of this study. But, the gender differences in this study were small when compared with previous studies. There were no significant differences in any of the subscales in the inpatient sample. Though this may be characteristic of Japan, further research to examine a larger sample and data from many institutions will be necessary.

### Limitations of this study

This study has several limitations. We did not gather the data of psychiatric patients in this study. Data collection for a group of psychiatric patients will be necessary in future study. When a cut-off point is set, utility is high for clinical application.

Also, our data was collected in only one area of Japan. This might include the possibility of local bias. Future research will include comparative studies of the data from several Japanese areas.

## Conclusion

We examined the validity and reliability of SCL-90-R(J). Almost of all of the correlation coefficients between the nine primary subscales and items were over .35. Cronbach's alpha coefficients were from .76 (Phobic Anxiety) to .86 (Interpersonal Sensitivity). Pearson's correlation coefficients between test-retest scores were from .81 (Psychoticism) to .90 (Somatization) for the outpatients and were from .64 (Phobic Anxiety) to .78 (Paranoid Ideation) for the students. Each of the nine primary subscales correlated well with their corresponding constructs in the MMPI. We confirmed the validity and reliability of SCL-90-R(J) for the measurement of individual distress. Because the nine primary subscales were consistent with the items of the original English version, this SCL-90-R(J) will be useful for comparative cross-culture study. Also, this psychological scale can evaluate multidimensional psychopathological aspects simultaneously, which makes it useful as a screening test for medical examination and treatment. For example, it will be useful for judging drug efficacy, such as SSRI for anxiety and depression.

## Competing interests

This study was supported by a grant from Smoking Research Foundation.

## Authors' contributions

MT conceptualized and designed the study, collected the data, performed analysis and interpreted the data, and drafted the manuscript. MS designed the study and translated the SCL-90-R to Japanese. MH collected the data. CK supervised the design of the study and the writing of the manuscript. All authors read and approved the final manuscript.
